# Induction of Tier 2 HIV-Neutralizing IgA Antibodies in Rhesus Macaques Vaccinated with BG505.664 SOSIP

**DOI:** 10.3390/vaccines12121386

**Published:** 2024-12-10

**Authors:** Justin C. Smith, Prabhu S. Arunachalam, Traci H. Legere, Lisa A. Cavacini, Eric Hunter, Bali Pulendran, Rama R. Amara, Pamela A. Kozlowski

**Affiliations:** 1 Department of Microbiology, Immunology and Parasitology, Louisiana State University Health Sciences Center, New Orleans, LA 70112, USA; jsmi68@lsuhsc.edu; 2 Institute for Immunity, Transplantation and Infection, Stanford University School of Medicine, Stanford, CA 94304, USA; prabhusa@arizona.edu; 3 Emory Vaccine Center, Division of Microbiology and Immunology, Emory National Primate Research Center, Emory University, Atlanta, GA 30329, USA; traci.legere@emory.edu; 4 Department of Medicine, University of Massachusetts Chan Medical School, Worcester, MA 01655, USA; lisa.cavacini@umassmed.edu; 5 Emory Vaccine Center, Department of Pathology and Laboratory Medicine, Emory National Primate Research Center, Emory University, Atlanta, GA 30329, USA; ehunte4@emory.edu; 6 Department of Microbiology and Immunology, Stanford University School of Medicine, Stanford, CA 94304, USA; bpulend@stanford.edu; 7 Department of Pathology, Stanford University School of Medicine, Stanford, CA 94304, USA; 8 Emory Vaccine Center, Department of Microbiology and Immunology, Emory National Primate Research Center, Emory University, Atlanta, GA 30329, USA; ramara@emory.edu

**Keywords:** HIV-1, polymeric IgA, neutralization, antibody, SOSIP, mucosal vaccine

## Abstract

Background: A goal of mucosal human immunodeficiency virus type 1 (HIV-1) vaccines is to generate mucosal plasma cells producing polymeric IgA (pIgA)-neutralizing antibodies at sites of viral entry. However, vaccine immunogens capable of eliciting IgA neutralizing antibodies (nAbs) that recognize tier 2 viral isolates have not yet been identified. Methods: To determine if stabilized native-like HIV-1 envelope (Env) trimers could generate IgA nAbs, we purified total IgA and IgG from the banked sera of six rhesus macaques that had been found in a previous study to develop serum nAbs after subcutaneous immunization with BG505.664 SOSIP and 3M-052 adjuvant, which is a TLR7/8 agonist. The neutralization of autologous tier 2 BG505 T332N pseudovirus by the IgA and IgG preparations was measured using the TZM-bl assay. Anti-SOSIP binding antibodies (bAbs) were measured by ELISA. Results: The IgG samples were found to have significantly greater levels of both nAb and bAb. However, after normalizing the nAb titer relative to the concentration of bAb, SOSIP-specific IgA purified from 2/6 animals was found to neutralize just as effectively as SOSIP-specific IgG, and in 3/6 animals, neutralization by the specific IgA was significantly greater. The more potent neutralization by IgA in these three animals was associated with a higher percentage of anti-SOSIP J chain-bound (polymeric) antibody. Conclusions: The parenteral vaccination of nonhuman primates with BG505.664 SOSIP generates HIV-1 tier 2 IgA nAbs in serum, including SOSIP-specific polymeric IgA, which appears to neutralize more efficiently than monomeric IgA or IgG. Mucosal delivery of this SOSIP or other stable Env trimers could generate locally synthesized polymeric IgA nAbs in mucosal tissues and secretions.

## 1. Introduction

HIV-1 is primarily transmitted at mucosal surfaces in the genital tract and rectum. The vaccine-induced synthesis of polymeric IgA (pIgA) anti-HIV-1 neutralizing antibodies (nAbs) by plasma cells in these mucosal tissues could be highly beneficial for preventing infection. pIgA antibodies have been shown to more robustly neutralize toxins and viral pathogens than monomeric forms of IgA and IgG, which predominate in serum [1,2,3,4,5,6,7,8]. The passive rectal administration of a recombinant dimeric IgA1 (dIgA1) expressing the variable domain of an HIV-1 nAb has also been reported optimal to the parental IgG1 nAb for preventing rectal simian-human immunodeficiency virus (SHIV) transmission in nonhuman primates (NHPs) [9]. Another class-switched HIV-1 nAb expressed as pIgA2 and intravenously infused into humanized mice provided better protection against vaginal HIV-1 infection than a monomeric IgA2 (mIgA2) version or the parental IgG1 nAb [10]. However, no HIV-1 vaccine has yet been shown to induce IgA nAbs in the sera or secretions of humans or NHPs. Theoretically, this should be possible because HIV-1 infection generates serum IgA antibodies that inhibit the in vitro infection of target cells by not only “easy to neutralize” tier 1 HIV-1 isolates [11,12] but also by “moderately difficult to neutralize” tier 2 strains [13,14], which better represent global circulating HIV-1 isolates [15].

Few HIV-1 Env immunogens have been able to elicit tier 2 nAbs. One of the more successful immunogens, BG505.664 SOSIP, is a stabilized gp120/gp41 disulfide-bonded trimeric protein [16] that maximizes the exposure of conformational neutralizing epitopes while hiding non-neutralizing determinants, such as the gp41 immunodominant domain [17]. We and others have shown that the subcutaneous (SC) immunization of rhesus macaques with BG505.664 SOSIP formulated with immune stimulatory complexes [18] or 3M-052 in nanoparticles [19] can generate tier 2 nAbs in serum and prevent rectal or vaginal infection with autologous tier 2 BG505 SHIV. Protection in these animals was presumably mediated by transudated serum IgG nAbs in the challenged tissue or local secretion because, as in humans [20], parenterally administered subunit vaccines typically do not generate vaccine-specific antibody-secreting IgA or IgG plasma cells in rectal or vaginal tissues of NHP [21,22].

SC immunization does generate systemic IgA responses. Therefore, in an effort to determine whether BG505.664 SOSIP could elicit tier 2 HIV-1 IgA nAbs, we evaluated IgA purified from the sera of six macaques that had previously been found to be protected against vaginal BG505 SHIV challenge after vaccination with 3M-052 adjuvanted-BG505.664 SOSIP [19]. Here, we show that on a per microgram basis, SOSIP-specific IgA can neutralize autologous tier 2 virus just as well or better than IgG, depending on how much pIgA Ab is present. These serum IgA nAbs probably did not significantly contribute to protection because their titers were dramatically lower than IgG. Nonetheless, the results demonstrate that the BG505.664 SOSIP immunogen can generate tier 2 IgA nAbs. If this or another stable trimer was administered by a mucosal route, it could potentially generate plasma cells secreting protective pIgA nAbs at sites of mucosal HIV transmission.

## 2. Materials and Methods

### 2.1. Animals

Frozen serum samples were obtained from 4–6.5-year-old female Indian-origin rhesus macaques that had received SC vaccinations with 3M-052-adjuvanted BG505.664 SOSIP alone or in addition to intravenous SIV gag-expressing heterologous viral vectors (HVV) in a previous study [19].

### 2.2. Purification of Total Serum IgA and IgG

IgG was purified from pooled sera using affinity column chromatography with recombinant Protein G Sepharose (GE Healthcare, Uppsala, Sweden). IgA was purified using a mixture of agarose-bound Peptide M and *Staphylococcus aureus* Superantigen-like protein 7 (SSL7) (Invivogen, San Diego, CA, USA). Bound IgG or IgA was eluted with 0.1 M glycine buffer, pH 2.5, and immediately neutralized with saturated Tris-base. The eluate was concentrated using Millipore Amicon Ultra15 centrifugal filters with a 50,000 MW cut-off (Sigma, St. Louis, MO, USA) and dialyzed in PBS. Total protein was measured using the bicinchoninic acid assay (Thermo Scientific, Waltham, MA, USA). The IgG and IgA preparations were diluted to 10 mg/mL in Dulbecco’s PBS containing Ca^+2^ and Mg^+2^ and 0.45 µm sterile-filtered using Corning Costar SpinX microcentrifuge filter tubes (VWR, Radnor, PA, USA). Some IgA samples were depleted of IgM by placing 500 µL of a 20% slurry of anti-human IgM antibody conjugated to agarose (Sigma) in the top chamber of a SpinX filter, centrifuging for 30 s at 15,000× *g*, removing the PBS diluent in the lower chamber, and then adding 500 µL IgA to the dry beads. After mixing for 1 h at 25 °C, the tube was centrifuged for 2 min, and the fluid in the lower chamber was retained.

### 2.3. ELISA for Immunoglobulin (Ig)

The total IgA, pIgA, IgG and IgM concentrations were measured by ELISA using Immulon 4 HBX microtiter plates (VWR) coated overnight at 4 °C with 100 µL per well of one of the following affinity-purified antibodies: 1 µg/mL clone IgA5-3B mouse anti-monkey α chain monoclonal antibody (mAb; Bio-Rad, Hercules, CA, USA), 0.5 µg/mL goat anti-monkey γ chain (Alpha Diagnostic International, San Antonio, TX, USA) or 0.5 µg/mL goat anti-human µ chain antibody (VWR). Plates were washed 4 times with PBS containing 0.05% Tween-20 (PBST) and blocked with 0.1% bovine serum albumin (BSA) in PBST. Serial dilutions of standard and samples diluted in block buffer were then added and allowed to react overnight at 4 °C. Standards were rhesus b12 mIgA, J chain-expressing rhesus b12 dIgA (both NHP Reagent Resource: NHPRR), rhesus IgG or rhesus IgM (both Rockland Immunochemicals, Pottstown, PA, USA). Plates were washed and developed with 0.1 µg/mL biotinylated goat anti-monkey α chain antibody (Rockland), clone CA1L mouse anti-human/rhesus J chain mAb (NHPRR), anti-human γ chain or anti-human µ chain antibody (SouthernBiotech, Birmingham, AL, USA) followed by 1/4000 neutralite avidin–peroxidase (SouthernBiotech) and tetramethylbenzidine (TMB). After recording absorbance at 370 nm in a SpectraMax5 plate reader, Softmax Pro software v5.0.1 (both Molecular Devices, San Jose, CA, USA) was used to construct standard curves and determine the concentration of Ig in each sample.

### 2.4. ELISA for Specific Binding Antibodies

Binding antibodies (bAbs) to BG505.664 SOSIP (a gift from Dr. John Moore, Weill Cornell Medical College), BG505.W6M.ENV.C1 gp120 or Clade C gp41 (Immune Technology Corp., New York, NY, USA) were measured using Immulon 4 plates coated overnight at 4 °C with 50 ng per well of protein in PBS, pH 7.4. After washing, the plates were loaded with serial dilutions of standard and sample. Following overnight reaction at 4 °C, plates were developed using biotinylated goat anti-monkey IgA or IgG (Rockland) or anti-human/rhesus J chain mAb, which was followed by the above reagents. After recording absorbance, concentrations of Ab were interpolated from standard curves.

In assays for J chain-bound polymeric anti-SOSIP bAbs, rhesus b12 dIgA was used as a standard. For SOSIP, gp120, or gp41 ELISAs, the standards were IgA or IgG that had been purified from the pooled sera of previously vaccinated, SHIV-infected macaques. The concentration of antibody in the standards was estimated by coating two rows of a plate with anti-monkey IgA or IgG antibody and other rows with antigen. 5-parameter curves were constructed with captured rhesus mIgA or rhesus IgG and then used to determine the concentration of IgA or IgG antibody in the standards. In SOSIP ELISAs, the IgA and IgG standards produced very similar 5-parameter curves, and the 50% effective concentration (EC_50_) for 100 ng/mL of each was almost identical (Supplementary Figure S1).

### 2.5. Pseudovirus Production

HEK 293T/17 cells (American Type Culture Collection, Manassas, VA, USA) were maintained at 37 °C in 5% CO_2_ in D10: Dulbecco’s Modified Eagle Medium containing 10% fetal bovine serum and penicillin, streptomycin, and L-glutamine (ThermoFisher Scientific, cat# 10378016). Pseudovirus was produced by co-transfecting 293T/17 cells in a T75 flask with 4 µg BG505 T332N expression plasmid (kindly provided by Dr. David Montefiori, Duke Medical School) and 8 µg pSG3∆Env [23] (AIDS Reagent Program) in Fugene 6 transfection reagent (Promega, Madison, WI, USA). After incubation for 72 h, the culture medium was harvested, clarified by 0.45 µm filtration, aliquoted and stored at −80 °C. The 50% tissue culture infectious dose (TCID_50_/_mL_) was then determined using TZM-bl luciferase indicator cells (AIDS Reagent Program) and the Spearman–Karber method.

### 2.6. Neutralization Assay

The nAb titers in the 10 mg/mL purified total IgA and IgG preparations were measured using the TZM-bl assay [24] with a 1/20 starting dilution of Ig (500 µg/mL) in D10 medium. The samples were added in duplicate to 96-well clear-bottom black plates and 3-fold serially diluted. An equal volume (50 µL) of medium containing 200 TCID_50_ BG505 T332N pseudovirus was added to all but 6 wells, which served as cell only “blank” controls. One row of wells received medium and virus alone (virus controls). After 1 h, 1 × 10^4^ TZM-bl cells in 100 µL medium with 30 µg/mL DEAE dextran was added to every well. After 48 h at 37 °C, the medium was removed, and 75 µL of 0.1% TritonX-100 in PBS was added. The plate was mixed for 5 min; then, 25 µL per well of Brite-Glo (Promega) was added. Relative Light Units (RLUs) were immediately recorded using a SpectraMax 5. After the subtraction of blanks, the RLUs were plotted against the dilutions of total IgA or IgG tested. The percentage of infectivity was determined by normalizing curves with Y = 0 as 0% and Y = the average of virus control wells as 100%. The 50% inhibitory dilution (ID_50_) was then determined by nonlinear regression analysis.

### 2.7. Statistics

GraphPad Prism v9.3 software (San Diego, CA, USA) was used to calculate neutralization titers (ID_50_) and perform statistical comparisons. The two-tailed unpaired *t*-test was used to compare IgA and IgG bAbs and neutralization titers. Correlations were analyzed using the two-tailed Spearman rank correlation test. The alpha value was set to 0.05 in all statistical analyses. Only *p* values ≤ 0.05 were considered significant.

## 3. Results

### 3.1. Strategy

**Figure 1:**
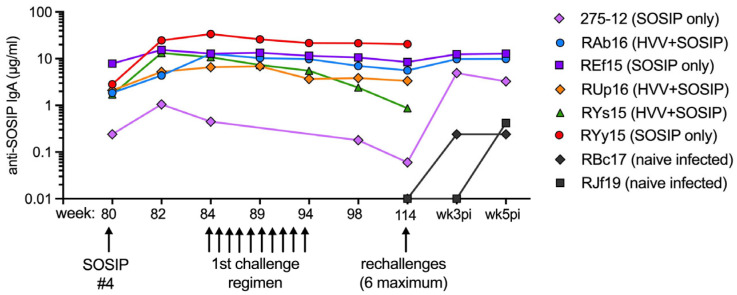
SOSIP-specific IgA in sera of vaccinated macaques before and after challenge. Female macaques were immunized by the SC route with BG505.664 SOSIP on wks 16, 24, 40 and 80 as described [19]. Half of the animals additionally received an IV immunization on wks 0, 8, and 36 with one of the following SIV gag-expressing heterologous viral vectors (HVVs): vesicular stomatitis virus, vaccinia virus, or adenovirus type 5, respectively. Shown are the concentrations of anti-SOSIP IgA antibody measured by ELISA after the 4th SOSIP immunization, throughout the 1st vaginal challenge regimen with BG505 SHIV, and on wks 3 and 5 post-infection (pi) following 1–6 rechallenges with the same SHIV. Lines that stop at wk 114 represent the 3 animals (RUp16, RYs15 and RYy15) that never became infected. Pre-immune levels of anti-SOSIP IgA bAbs were analogous to those shown on wk 114 for naive control macaques.

To determine if IgA nAbs could be generated by vaccination with a stable HIV-1 SOSIP immunogen, we decided to test IgA purified from the banked sera of rhesus macaques that had been found to develop tier 2 serum nAbs after SC immunization with 3M-052-adjuvanted BG505.664 SOSIP in a previous study [19]. These NHP had been immunized four times with SOSIP, either alone or in combination with intravenous (IV) heterologous viral vectors (HVVs) expressing SIV gag. On wk 84, 4 wks after the last SOSIP immunization, the animals were vaginally challenged at weekly intervals with BG505 SHIV a total of 10 times (Figure 1). Animals with serum nAb titers > 319 were protected and re-challenged 20 wks later with BG505 SHIV. By this time (wk 114), nAb titers in the majority of animals had declined, and most became infected unless they had SIV gag-specific T cells as a result of the HVV-gag immunizations [19].

For the current study, 6 NHPs that had anti-SOSIP serum IgA bAbs that were at least 10-fold above baseline and serum nAb titers > 319 on wk 82 were selected. The amount of wk 82 sera was very limited. However, after the 4th SOSIP immunization, IgA responses were quite durable in most of these animals (Figure 1). Therefore, 1 mL of wk 82 and wk 84 sera was pooled with 0.5 mL sera collected at subsequent time points shown in Figure 1. We included 0.5 mL of wk 3 post-infection (pi) and wk 5 pi sera for the 3 animals (275-12, RAb16, REf15) that became infected during the re-challenge regimen (Figure 1). IgA was isolated using affinity chromatography with Peptide M + SSL7. The total IgG in the same serum samples was also purified using Protein G. For controls, IgA and IgG were purified from the pooled sera of 10 naive macaques and from 2 pools of wk 3 pi + wk 5 pi sera from SHIV-infected nonvaccinated controls (each n = 4). The IgA and IgG solutions were adjusted to 10 mg/mL and sterile filtered. These solutions are roughly 10× for IgA and 1× for IgG based on the finding that the serum of female rhesus macaques contains mean total IgA and IgG concentrations of 1.4 mg/mL and 14 mg/mL, respectively (Supplementary Figure S2).

### 3.2. Lack of Significant IgG Contaminant in IgA Preparations

To confirm that the purified IgA or IgG samples were not contaminated with significant amounts of other Igs, ELISA with highly specific anti-IgA, -IgG or -IgM capture or detector antibodies (Supplementary Figure S3) was used to measure the total IgA, IgG and IgM in each solution (Table 1).

Less than 0.7% of the Ig in IgG solutions was IgA or IgM (Table 1). IgA preparations from the vaccinated NHP were >95% IgA and had <0.5% IgG. The major contaminant in the IgA samples was IgM (Table 1). However, this was not considered a significant problem because only RUp16 and RYs15 had been found to have anti-SOSIP IgM bAbs in sera [19], and the IgM in the IgA samples from these two animals was successfully removed using anti-IgM beads (Table 1).

These data indicate that the IgA samples were not contaminated with significant amounts of IgG, which was critical because very strong anti-SOSIP IgG responses had been observed in the immunization study [19,25].

### 3.3. Neutralization by Purified IgA and IgG

**Figure 2:**
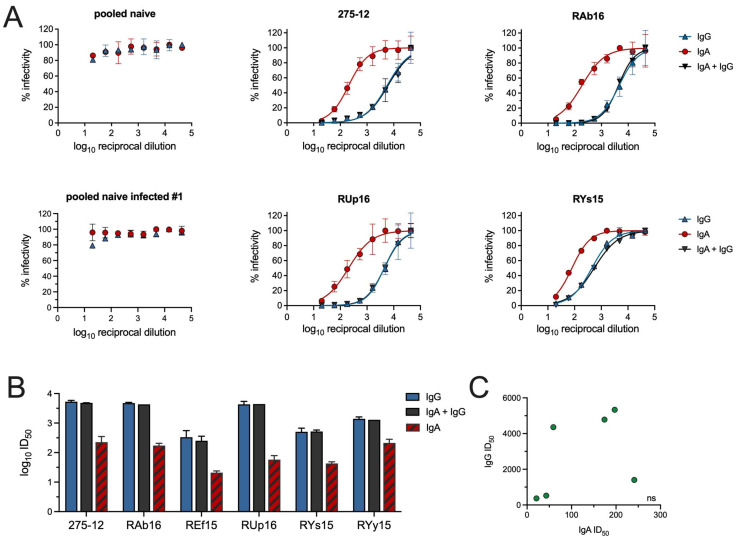
Titers of nAbs in IgA and IgG preparations. (**A**) Shown are representative normalized infectivity curves obtained in neutralizing assays with BG505 T332N pseudovirus and the 10 mg/mL total IgA or IgG preparations purified from sera of naive and vaccinated rhesus macaques before and after challenge. Equivalent amounts of the IgA and IgG were tested alone or in combination using a 1/20 starting dilution. No nAbs were detected in IgA or IgG samples from naive animals. Error bars denote SD. (**B**) The average nAb titer determined from 2–3 assays with each IgA and IgG sample from vaccinated animals are presented with SD. For every animal, the ID_50_ for IgG or IgA + IgG was found to be significantly greater than the ID_50_ for IgA alone (all *p* < 0.05 by unpaired *t*-test). (**C**) The nAb titers for IgA and IgG from vaccinated animals were not correlated using the Spearman rank correlation test (*p* = 0.1750).

Neutralizing antibodies (nAbs) against tier 2 BG505 T332N pseudovirus [25] were next measured in the purified IgA and IgG samples using the TZM-bl assay. Importantly, none of the control IgA or IgG samples purified from unvaccinated animals before or after challenge were found to neutralize (Figure 2A), indicating that the purification procedures did not introduce contaminants capable of inhibiting viral replication.

All of the IgA samples from vaccinated animals were able to neutralize with ID_50_ ranging from 20 to 241 (Figure 2B). Significantly greater neutralization was observed by IgG, which had ID_50_ from 365 to 5332 (Figure 2B). The ID_50_ values for IgA and IgG samples were not correlated (Figure 2C), supporting a lack of IgG contaminant in the IgA samples. Equal amounts of combined purified total IgA + IgG from each animal were also tested for neutralization. There was no evidence of additive or synergistic effects; the ID_50_ values for total IgA + IgG were virtually identical to those obtained with IgG alone (Figure 2B). Although the IgG nAb titers were much higher, these data clearly demonstrate that tier 2 IgA nAbs can be generated in NHP with the BG505.664 SOSIP protein.

### 3.4. Anti-SOSIP bAb Concentrations

**Figure 3:**
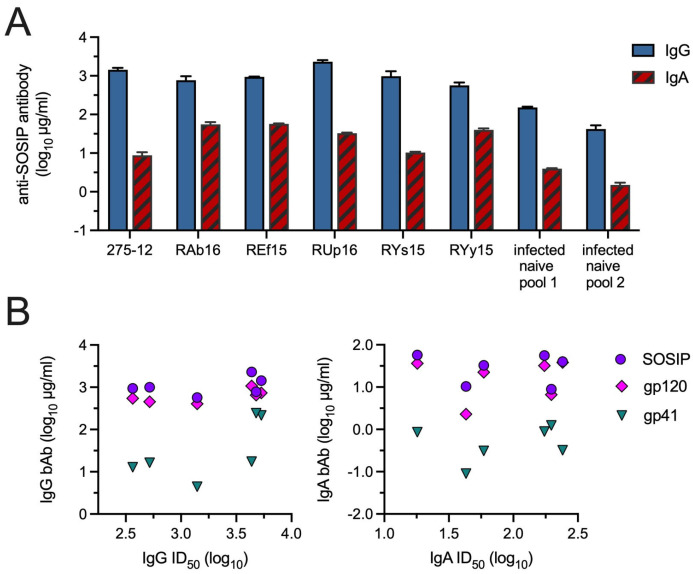
Anti-Env bAbs in IgA and IgG samples. Concentrations of IgA and IgG specific for SOSIP, gp120 or gp41 were measured by ELISA. (**A**) Anti-SOSIP IgA and IgG bAbs. Results are the mean + SD of 2–3 assays. The IgG purified from every animal contained significantly greater concentrations of anti-SOSIP bAb compared to their IgA (all *p* < 0.05 by unpaired *t*-test). (**B**) Comparison of IgG or IgA nAb titers with concentrations of anti-SOSIP, -gp120 and -gp41 bAb concentrations. Using the Spearman rank test, no significant correlations were found (all *p* > 0.05).

We suspected that greater neutralization by IgG might simply be due to a higher concentration of anti-SOSIP IgG bAb in these preparations. The concentrations of anti-SOSIP bAbs in the IgA and IgG solutions were therefore quantified using ELISA. Levels of SOSIP-specific bAb in IgG samples from the vaccinated NHP ranged from 570 to 2310 µg/mL and were indeed significantly higher than those in the IgA preparations, which contained only 9–57 µg/mL bAb (Figure 3A).

Concentrations of anti-SOSIP bAbs in the samples did not, however, correlate with nAb titers (Figure 3B). There were also no correlations between titers of nAb and concentrations of anti-gp120 or anti-gp41 bAbs in the IgA and IgG samples (Figure 3B). The lack of associations between nAb titers and Env bAb concentrations is consistent with other studies [18,19].

### 3.5. Neutralization Capacity of the Anti-SOSIP IgA and IgG

**Figure 4:**
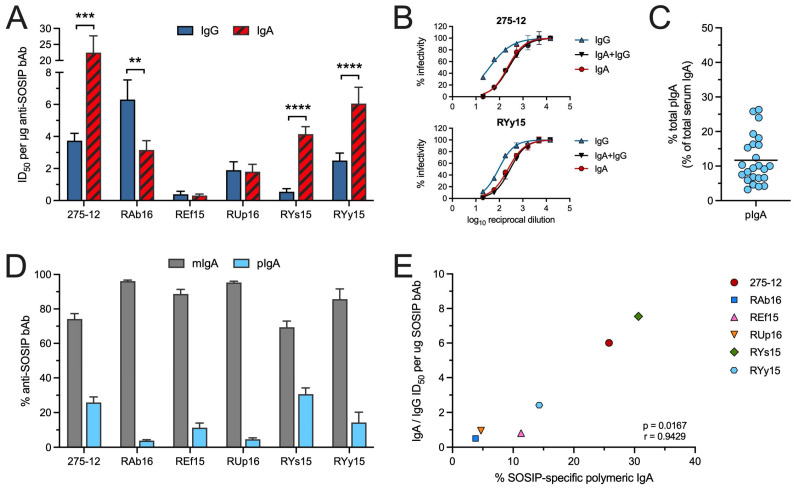
Neutralizing capacity of the SOSIP-specific IgA and IgG. (**A**) The ID_50_ for each purified IgA or IgG sample was divided by the concentration of anti-SOSIP bAb to compare the neutralization efficiency of the SOSIP-specific IgA and IgG. Shown are the mean + SD for the replicate assays. ** *p* < 0.01, *** *p* < 0.001, **** *p* < 0.0001 by unpaired *t*-test. (**B**) Infectivity curves obtained for 275-12 IgA and RYy15 IgA in neutralization assays with an equivalent amount of anti-SOSIP IgG. RYs15 IgA combined with an equal amount of SOSIP-specific IgG also produced an ID_50_ that was equivalent to RYs15 IgA alone. (**C**) The % of total polymeric IgA in serum of 14 rhesus macaques was calculated after measuring the concentrations of total IgA and J chain-bound IgA by ELISA. (**D**) The % of polymeric anti-SOSIP IgA was determined after measuring anti-SOSIP J chain-bound Ab and anti-SOSIP IgA in purified IgA samples by ELISA. (**E**) The fold difference between IgA and IgG neutralization capacities (ID_50_ per µg of SOSIP-specific bAb) was compared to the % of anti-SOSIP pIgA bAb in the IgA samples purified from each animal and found to be significantly correlated using the Spearman rank test.

To compare the relative efficiency of the anti-SOSIP bAb in each IgA and IgG preparation to neutralize, the ID_50_ was normalized relative to the anti-SOSIP bAb concentration. This revealed that on a per µg basis, anti-SOSIP IgG in only 1/6 animals neutralized the T332N pseudovirus more efficiently than their anti-SOSIP IgA (Figure 4A). In the remaining five animals, two had an ID_50_ per µg of SOSIP-specific IgA that was similar to the ID_50_ per µg of SOSIP-specific IgG, and three (275-12, RYs15 and RYy15) had anti-SOSIP IgA that neutralized to a significantly greater extent (2.4–7.5-fold) than their anti-SOSIP IgG (Figure 4A).

For these three animals, we tested neutralization by their IgA + IgG after diluting the IgG to contain a concentration of anti-SOSIP bAb equivalent to that in the IgA. In contrast to results with equal concentrations of total IgA + IgG, the ID_50_ in these assays was virtually identical to that obtained with the IgA alone (Figure 4B), supporting the greater ability of the SOSIP-specific IgA to neutralize.

Superior neutralization by mIgA antibodies compared to IgG is certainly not without precedence. The conversion of several anti-HIV human IgG1 nAbs to human mIgA2 or mIgA1 has been reported to increase neutralizing potency [26,27,28]. However, we considered that greater neutralization by the SOSIP-specific IgA in these three animals might simply be due to the presence of higher avidity polymeric nAb. The percentage of pIgA in the circulation of rhesus macaques has never been quantified, but high molecular weight IgA corresponding to dimers has been shown to be present in rhesus macaque sera [29]. Using ELISA and a recently developed anti-human/rhesus J chain mAb [30], we determined that 10–30% of the total serum IgA in rhesus macaques was bound by the J chain, and, hence, polymeric (Figure 4C).

To determine what percentage of the anti-SOSIP IgA in the purified IgA samples was polymeric, we used the anti-J chain antibody as secondary in the SOSIP ELISA. The results indicated that a greater fraction of the anti-SOSIP IgA in 275-12, RYs15 and RYy15 was polymeric compared to the other animals in the study (Figure 4D). There were no correlations between nAb titers and concentrations of SOSIP-specific pIgA bAb. However, an increased proportion of anti-SOSIP polymeric antibody in the IgA samples was significantly associated with a greater ability to neutralize when compared to SOSIP-specific IgG (Figure 4E). These results demonstrate that Env-specific pIgA antibodies can be generated by vaccination with BG505.664 SOSIP, and they suggest that the pIgA may be more effective than mIgA or IgG Abs for neutralization.

## 4. Discussion

To our knowledge, this is the first report of an HIV-1 Env immunogen capable of generating tier 2 IgA nAbs in primates. Although these IgA nAbs were in serum, and likely derived from the bone marrow [23,31], we show that as in humans [32], 10–30% of the serum IgA in rhesus macaques is polymeric, and the BG505 SOSIP-induced pIgA Abs in serum may neutralize virus more effectively than monomeric IgA or IgG. Of course, HIV-1 vaccines should aim to generate IgG nAbs, rather than IgA, in blood. Levels of serum IgA are considerably lower due to a much shorter half-life [23], and circulating pIgA Abs transudate very poorly into mucosal tissues [23]. The induction of IgA nAbs in rectal and genital tract mucosal tissues, where plasma cells primarily produce pIgA, would be far more advantageous for preventing the sexual transmission of HIV-1.

Harnessing humoral immune effectors in both the systemic and mucosal immune systems would likely be optimal for protection against HIV-1. Synergy between serum IgG and mucosal pIgA nAbs has been illustrated in NHP [33]. The IV infusion of an HIV-1 IgG1 nAb followed by the rectal application of a dIgA2 nAb with the same specificity was shown to prevent rectal SHIV infection, although neither of these nAbs on their own were able to do so [33]. Combining the intramuscular (IM) immunization route with a mucosal vaccine delivery route should elicit both systemic IgG and mucosal IgA Abs, but the mucosal route should be capable of generating Ab responses in both the male and female genital tract as well as in the rectum. To date, only the intranasal (IN) mucosal immunization route has been demonstrated to induce local IgA Abs or IgA antibody-secreting plasma cells at all of these sites in primates [29,34,35,36]. IM priming followed by IN boosting of NHP with an HIV gp41 virosome-based vaccine that induced mucosal antibodies was shown to be effective for preventing vaginal SHIV infection, although IM immunization was not [37]. Importantly, IN immunization can also generate beneficial antiviral gag-specific T cells in the female genital tract and rectum [34].

There are several limitations of this study. First, we could not measure the affinity of the anti-SOSIP IgA bAbs using surface plasmon resonance because the vast excess of nonspecific IgA in the purified total IgA preparations caused too much interference. Second, the SOSIP epitope specificity of IgA Fabs could not be evaluated by electron microscopy polyclonal epitope mapping (EMPEM) [38] because rhesus IgA is resistant to cleavage by human IgA1 proteases [39], and there is no known method for cleaving the native IgA of this species into Fabs. We also had too little IgA for fractionation of the polymeric IgA. Finally, we could not determine if the IgA might have had greater neutralization breadth than the IgG, nor could we map neutralizing epitopes using mutant pseudoviruses [40,41], because the amount of IgA isolated was insufficient to perform additional neutralizing assays.

It is known that the IgG nAbs induced by BG505.664 SOSIP target regions of gp120 that are not shielded by N-linked glycans [40] and others [25] have shown that epitopes proximal to the C3/465 “glycan hole” are most frequently targeted by the serum IgG nAbs in the macaques used here. Other neutralization determinants were found to be the 241/289 glycan hole and, in the case of RAb16 and RYy15, an epitope in V1. The absence of a clear additive effect in our neutralization assays with both IgA and IgG suggests that the nAbs in these preparations may target the same neutralizing determinants. If so, then the IgA nAbs, in addition to the IgG, are likely to be strain-specific because the 465 glycan hole is unique to BG505.

## 5. Conclusions

In summary, we show that tier 2 IgA nAbs can be generated with BG505.664 SOSIP. Although improvements to this and other SOSIPs are needed to generate broadly nAbs, there is no reason to believe that BG505.664 SOSIP would not similarly generate plasma cells secreting pIgA nAbs in a mucosal tissue if it was efficiently internalized with adjuvant at an appropriate mucosal surface. Future studies will be required to determine if this observed tier 2 IgA nAb induction utilizing SOSIP is indeed feasible when administered mucosally.

## Figures and Tables

**Table 1:** Immunoglobulin content of purified IgG and IgA samples.

	% IgG	% IgA	% IgM
**IgG preparations:**			
275-12	99.5	0.2	0.3
RAb16	99.4	0.1	0.5
REf15	99.5	0.2	0.3
RUp16	99.9	0.0	0.1
RYs15	99.7	0.1	0.2
RYy15	99.5	0.3	0.2
Pooled naive	99.6	0.1	0.3
Pooled naive/infected #1	99.3	0.2	0.5
Pooled naive/infected #2	99.1	0.3	0.6
**IgA preparations:**			
275-12	0.3	98.8	1.0
RAb16	0.3	96.3	3.5
REf15	0.1	95.8	4.3
RUp16	0.1	99.7	0.1
RYs15	0.4	99.6	0.1
RYy15	0.2	98.3	1.5
Pooled naive	0.9	94.9	4.2
Pooled naive/infected #1	0.4	97.9	1.7
Pooled naive/infected #2	0.2	97.7	2.1

Total IgG, IgA and IgM concentrations were quantitated by sandwich ELISA using highly-specific capture or detector antibodies, as described in Materials and Methods. After summing the concentrations, the % of IgG, IgA and IgM in each preparation was calculated.

## Data Availability

Data are contained within the article or Supplementary Materials.

## Supplementary Materials

The following supporting information can be downloaded at https://www.mdpi.com/article/10.3390/vaccines12121386/s1, Supplementary Figure S1: Performance of IgA and IgG standards in the SOSIP ELISA; Supplementary Figure S2: Total IgA and IgG concentrations in serum of rhesus macaques; Supplementary Figure S3: Specificity of antibodies used to measure immunoglobulin concentrations.
