# Supplementary material for: Induction of Tier 2 HIV-Neutralizing IgA Antibodies in Rhesus Macaques Vaccinated with BG505.664 SOSIP
Source: Vaccines (Basel). 2024 Dec 10;12(12):1386. doi: 10.3390/vaccines12121386 (PMC11680376; doi:10.3390/vaccines12121386)
Supplement: Supplementary file 1 [file vaccines-12-01386-s001.zip › vaccines-3331199-supplementary.pdf]

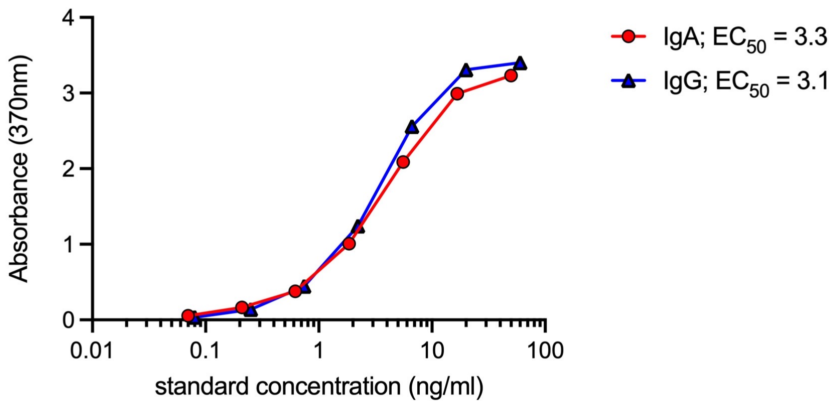

Supplementary Figure S1. Performance of IgA and IgG standards in the SOSIP ELISA. Total IgA and IgG purified from pooled serum of vaccinated rhesus macaques that had succumbed to SHIV infection in previous studies were used as standards in all HIV Env-based ELISAs. Shown are representative dilution curves and EC_{50} for each calibrated standard in the SOSIP ELISA.

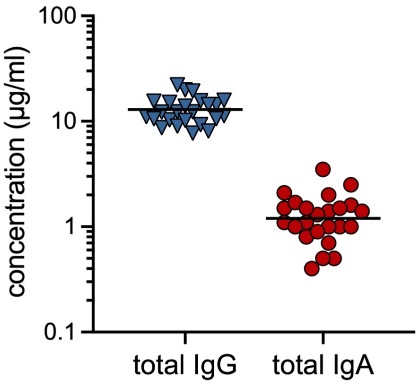

Supplementary Figure S2. Total serum IgA and IgG in rhesus macaques. Comcentrations of IgA and IgG were measured in serum of 24 female macaques by ELISA. Bars denote arithmetic means.

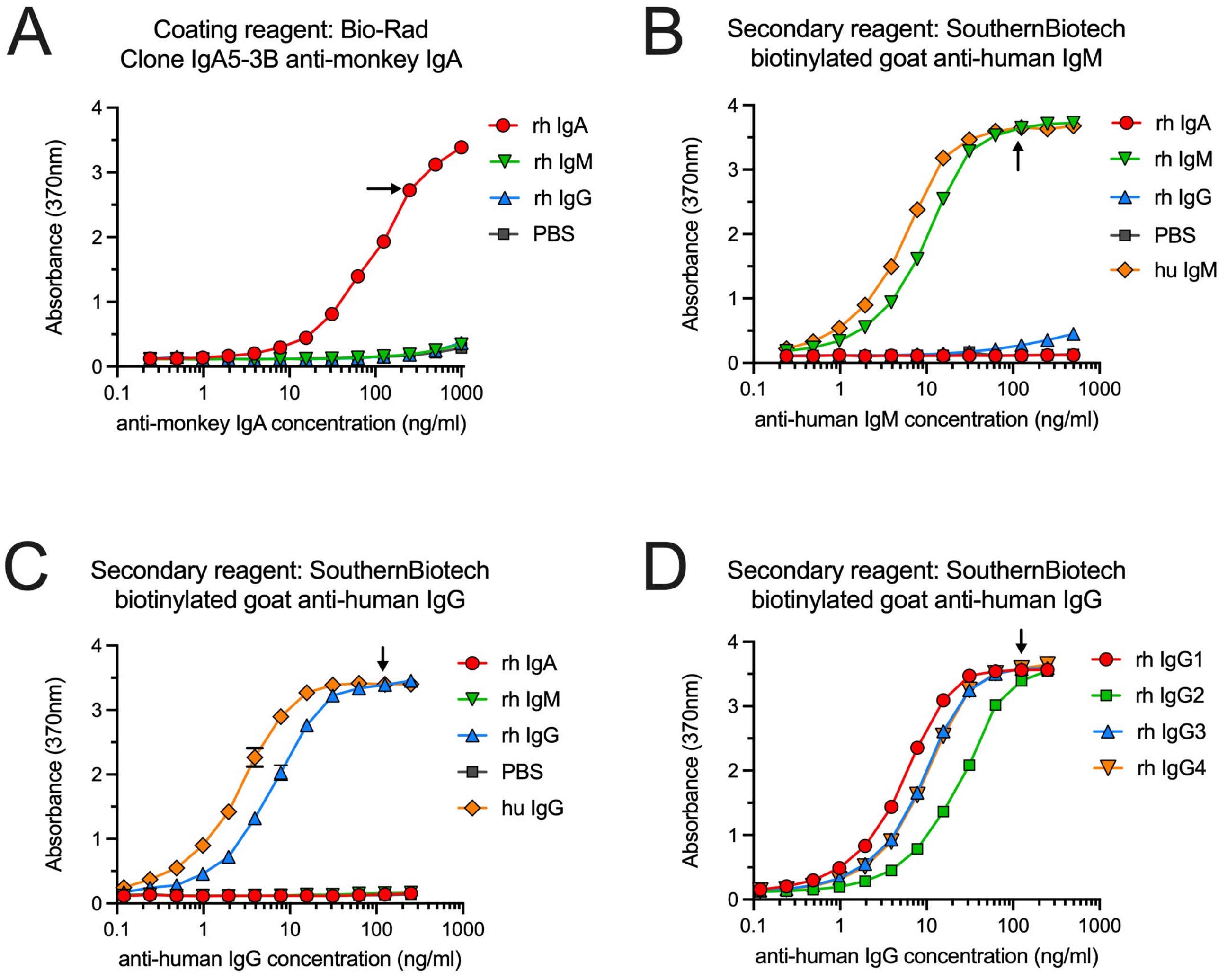

Supplementary Figure S3. Specificity of antibodies used to measure Ig concentrations by ELISA was evaluated by coating different rows of a microtiter plate with 1μg/ml of **(A-C)** rhesus IgA, IgG or IgM or **(D)** rhesus IgG1, IgG2, IgG3 or IgG4 (NHP Reagent Resource). After washing and blocking, serial dilutions of IgA-, IgG- or IgM-specific biotinylated antibody were added to every well. Following a 1h incubation at 37˚C, the plates were developed with avidin-peroxidase and TMB. After 20-30 minutes, absorbance was recorded.
